# Spectrum of Cancers and Their Prognosis Among Patients With Myotonic Dystrophy

**DOI:** 10.1001/jamanetworkopen.2025.26894

**Published:** 2025-08-13

**Authors:** Shahinaz M. Gadalla, Timothy S. McNeel, William Wheeler, Rotana Alsaggaf, Bilal Hameed, Soundarya Avantsa, Muzzammil Ahmadzada, Ruth M. Pfeiffer

**Affiliations:** 1 Clinical Genetics Branch, Division of Cancer Epidemiology and Genetics, National Cancer Institute, National Institutes of Health, Bethesda, Maryland; 2 Information Management Services, Inc, Calverton, Maryland; 3 Department of Neurology, Yale School of Medicine, New Haven, Connecticut; 4 Stanford University School of Medicine, Stanford, California; 5 Biostatistics Branch, Division of Cancer Epidemiology and Genetics, National Cancer Institute, National Institutes of Health, Bethesda, Maryland

## Abstract

This cohort study examines the distribution and survival outcomes of cancer types among patients with myotonic dystrophy, highlighting increased risks for certain cancers compared with the US general population.

## Introduction

Myotonic dystrophy (*dystrophia myotonica;* DM) is a multisystem disease caused by an autosomal dominant unstable nucleotide repeat expansion in *DMPK* or *CNBP*. Patients with DM have higher risk of endometrial, ovarian, eye, thyroid, brain, and colon cancers than the general population.^1^ To further understand cancer burden among patients with DM, we compare the distribution of cancer types and outcomes among patients with DM vs cancer patients in the US general population.

## Methods

Using the cancer cohort of the Surveillance, Epidemiology, and End Results (SEER)–Medicare linked database, we identified patients with DM (*ICD-9* code 359.21) with at least 1 Medicare inpatient claim, or 2 claims from other Medicare sources at least 30 days apart between 1992 and 2016. Cancer records were available from 1992 to 2015. Patients with first primary cancers from SEER-12 and SEER-17 comprised the comparison cohort. We followed STROBE guidelines. The study used deidentified data and was not considered human participants research by the National Institutes of Health.

We calculated standardized proportion ratios (PRs) to compare the distribution of first malignant primary cancers in patients with DM and SEER. We used Cox proportional hazard models to compare post-cancer survival between the DM and SEER populations, adjusted for age, sex, and stage at cancer diagnosis. *P* ≤ .01 (2-sided) was considered significant. Calculations were performed between July 22 and November 5, 2024, using SAS, version 9.4 and R, version 4.4.2 (eMethods in Supplement 1).

## Results

We identified 510 patients with DM with 633 primary cancers (behavior codes 0-3); 259 (50.8%) received Medicare for disability. Of them, 381 (74.7% vs 82.9% in SEER) had 1 malignant tumor (code 3) and 66 had at least 2 (12.9% vs 9.1% in SEER). Median age at first cancer diagnosis was 63.2 (range, 21.8-97.7) years.

The composition of first malignant primary cancers found in 430 patients with DM differed significantly from that reported in SEER patients. Compared with expected proportions based on SEER, patients with DM were more likely to have cancers of the endocrine glands (PR, 2.4; 95% CI, 1.6-3.2), gynecologic organs (PR, 1.5; 95% CI, 1.1-1.9), and hematologic system (PR, 1.9; 95% CI, 1.4-2.4) but less likely to have respiratory cancers (PR, 0.6; 95% CI, 0.4-0.8) (Table). Among these patients, 29 of 30 endocrine cancers (96.7%) were of the thyroid gland, 33 of 37 gynecologic cancers (89.2%) were endometrial or ovarian, and hematologic malignant neoplasms were split equally between myeloid and lymphoid subtypes (23 of 45 [51.1%] were myeloid).

**Table.** Standardized Proportion Ratios of Malignant Tumors Among Patients With Myotonic Dystrophy Compared With Cancer Patients in SEER Diagnosed From 1992 to 2015

Cancer site^a^	No. observed	No. expected	PR (95% CI)	*P* value
Head and neck	12	16.2	0.7 (0.4-1.2)	.30
Endocrine glands	30	12.4	2.4 (1.6-3.2)	<.001
Female breast	50	64.3	0.8 (0.6-0.97)	.04
Gynecologic organs	37	25.1	1.5 (1.1-1.9)	.007
Gynecologic organs (excluding cervix)	35	21.1	1.7 (1.1-2.2)	.001
Hematologic	45	23.3	1.9 (1.4-2.5)	<.001
Myeloid	23	10.8	2.1 (1.3-3.0)	<.001
Lymphoid	22	12.1	1.8 (1.1-2.6)	.002
Lower digestive system	49	40.5	1.2 (0.9-1.5)	.20
Upper digestive system^b^	20	12.4	1.6 (1.0-2.3)	.03
Male genitalia	68	71.9	0.9 (0.8-1.1)	.60
Respiratory tract	29	51.2	0.6 (0.4-0.8)	.002
Skin	24	27.9	0.8 (0.5-1.2)	.50
Urinary system	32	31.4	1.0 (0.7-1.3)	.90

^a^ Only cancer sites with more than 11 patients were reported to accommodate the SEER-Medicare restriction in reporting small numbers.

^b^ Includes esophagus, stomach, and small intestine.

The PR of gynecologic cancers was significantly higher among younger women with DM than older (PR, 2.4 vs 1.1 for age <50 vs ≥50 years, respectively; *P* for heterogeneity = .004), even after excluding cervical cancer (PR, 3.5 vs 1.2, respectively; *P* for heterogeneity < .001). The PR for endocrine cancers was significantly higher among men with DM than women (PR, 4.3 vs 1.7 among men vs women, respectively; *P* for heterogeneity = .002).

Compared with SEER patients, patients with DM and cancer had a lower risk of all-cause (adjusted hazard ratio [aHR], 0.8; 95% CI, 0.7-0.9; *P* = .004) and cancer-specific (aHR, 0.5; 95% CI, 0.4-0.7; *P* < .001) mortality (Figure). Lower cancer-specific mortality risk among patients with DM was noted for most sites and was significant for hematologic malignant neoplasms (aHR, 0.4; 95% CI, 0.2-0.8; *P* = .008).

**Figure.**
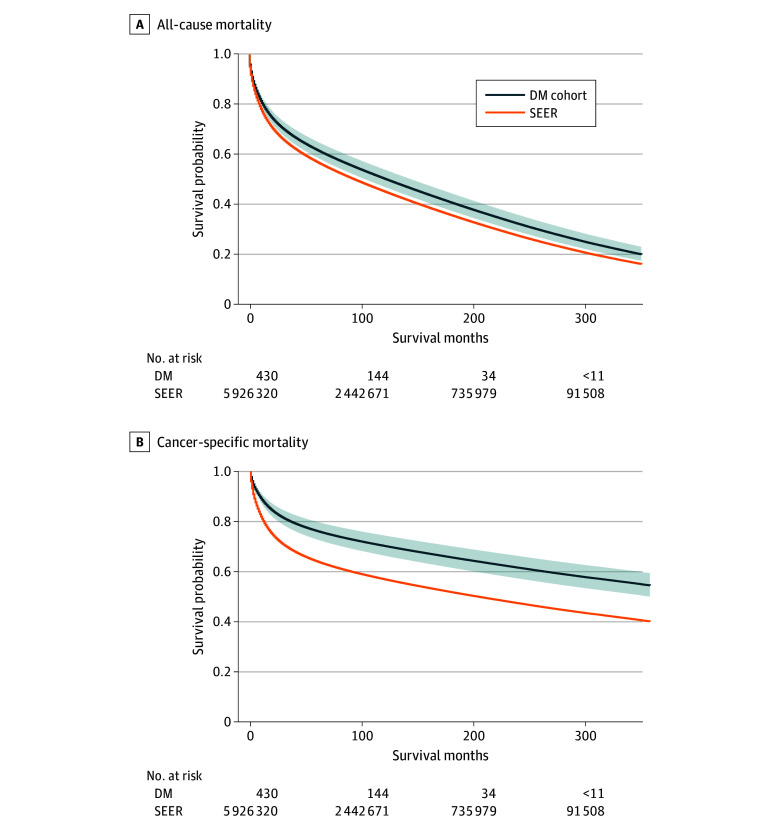
Adjusted Survival Probabilities After Cancer Diagnosis Among Patients With Myotonic Dystrophy and in SEER Curves are generated from Cox proportional hazard models; shaded areas represent 95% CIs. DM indicates myotonic dystrophy; SEER, Surveillance, Epidemiology, and End Results.

## Discussion

Cancer is the third leading cause of death among patients with DM.^2^ In this study, patients with DM with cancer had better prognosis than patients with cancer from the general population. This finding, if validated after considering possible residual confounders such as comorbidities and access to care, suggests that current cancer treatment for DM might be acceptable.

This study adds to evidence supporting that DM is an inherited cancer susceptibility syndrome.^3,4,5,6^ However, not having an *ICD* diagnostic code that differentiates between DM1 and DM2 diagnoses is limiting. Future research is warranted to define effective cancer screening strategies for patients with DM given the differences in cancer distribution in this population vs the general population.

## Notes

Supplement 1.
eMethods.

Supplement 2.
Data Sharing Statement
