# Error in the Figure

**DOI:** 10.1001/jamanetworkopen.2025.42899

**Published:** 2025-10-14

In the Research Letter titled “Spectrum of Cancers and Their Prognosis Among Patients With Myotonic Dystrophy,”^1^ published August 13, 2025, the Figure contained a reporting error; in panels A and B, the numbers at risk at 300 months for the myotonic dystrophy group were mistakenly reported below the approved threshold for reporting. Those values have been changed to <11. This article has been corrected.^1^
