# Supplementary material for: Spectrum of Cancers and Their Prognosis Among Patients With Myotonic Dystrophy
Source: JAMA Netw Open. 2025 Aug 13;8(8):e2526894. doi: 10.1001/jamanetworkopen.2025.26894 (PMC12351407; doi:10.1001/jamanetworkopen.2025.26894)
Supplement: Supplement 1. — eMethods. [file jamanetwopen-e2526894-s001.pdf]

# Supplemental Online Content

Gadalla SM, McNeel TS, Wheeler W, et al. Spectrum of cancers and their prognosis among patients with myotonic dystrophy: cancer in myotonic dystrophy. *JAMA Netw Open.* 2025;8(8):e2526894. doi:10.001/jamanetworkopen.2025.26894

**eMethods. Supplemental Method**

This supplemental material has been provided by the authors to give readers additional information about their work.

© 2025 Gadalla SM et al. JAMA Network Open.

## Supplemental Method

*DM Study Population*

Patients with myotonic dystrophy (DM) (*International Classification of Diseases, Ninth Edition* [ICD-9] code 359.21) records between 1992 and 2016 were ascertained from the cancer cohort of the Surveillance, Epidemiology, and End Results (SEER)-Medicare linked database (https://healthcaredelivery.cancer.gov/seermedicare/overview/). We included DM patients who were entitled to Medicare coverage because of either reaching age 65 years or receiving disability insurance benefits but excluded those with end stage renal disease (because of its known link with certain cancers).

*Cancer Ascertainment in DM Study Population*

From available cancer records (1992-2015), we identified all incident primary cancers in the DM cohort that were reported to SEER. Cancers identified only from death certificates or autopsies were excluded. Recorded cancer sites based on ICD-O-3 (*International Classification of Diseases for Oncology, Third Edition*) were combined into organ systems to accommodate the SEER-Medicare restriction in reporting small numbers (N<11).

*General population data*

We used data from SEER-12 (1992-1999) and SEER-17 (2000-2015) for population comparisons to capture cancers reported before and after the year 2000, respectively. The analyses were restricted to first primary cancers, diagnosed at age ≥20 years (as no childhood cancers reported in DM patients) and with ICD-O-3 behavior=3 (ie, malignant, to avoid detection bias related to cancer screening practice or adherence).

© 2025 Gadalla SM et al. JAMA Network Open.

*Statistical analysis*

To assess differences in the overall distribution of cancers in the DM and general SEER population we fit a multinomial regression model that included "population (DM vs SEER)" as the predictor to all cancer sites combined, additionally adjusted for age, and sex.

To compare the distribution of different cancers in the DM and general population we calculated standardized proportion ratios (PRs), ie, the ratios between observed proportions of cancers at specific sites in cancer patients with DM and the proportion expected if these patients were part of SEER. PRs were calculated overall and stratified by reason for Medicare enrollment (retirement or disability insurance), sex, or age (< 50 or ≥50 years). For this calculation cancers in the DM population were restricted to first primaries only with behavior code 3. Expected proportions were obtained from a multinomial model fit to the SEER cancer population, adjusted for age (20-<35, 35-<50, 50-<60, 60-<70, ≥70 years), sex, and race and ethnicity. Corresponding 95% CIs were calculated using a bootstrap with 5000 resampling. Two-sided *P* values for the hypothesis of specific proportion ratios being equal to one were computed using a bootstrap (details below).

To quantify differences in all-cause (primary outcome) and cancer-specific mortality (secondary outcome), we fitted Cox proportional hazard models and calculated hazard ratios (HRs) and 95% CIs to cancer patients with DM and those diagnosed in SEER combined and included a term for "population" to quantify differences. Time since diagnosis was used as the underlying time scale. The models were additionally adjusted for age (in the same categories as above), sex, and stage at cancer diagnosis.

© 2025 Gadalla SM et al. JAMA Network Open.

All tests were 2-sided, with statistical significance defined as $P \leq .01$ to lessen the chance of testing-related false discovery. All calculations were performed in SAS version 9.4 (SAS Institute) and R version 4.4.2 (R Project for Statistical Computing).

*Standardized prevalence ratio calculation*

Let $Y = (Y_0, Y_1, Y_2, \ldots. Y_K)$ denote the different cancer groups where $Y_i = 1$ if the person is a case of type $i$ and 0 otherwise, $i = 0, \ldots, K$. We use polytomous logistic regression to compare each case group with a chosen reference group, $Y_0$, by modeling:

$$\mathrm{P}(Y_i = 1|X) = \frac{exp(\theta_{i1}+\theta_{i2}X_1+\cdots\theta_{im}X_m)}{1+\sum_{k=1}^{K} exp(\theta_{k1}+\theta_{k2}X_1+\cdots\theta_{km}X_m)}, \text{ i=0,…,K} \quad (1)$$

for the covariate vector $X = [1, X_1, \ldots, X_m]$, that includes a one for the intercept term. As $\mathrm{P}(Y_0 = 1|X)+\ldots \mathrm{P}(Y_K = 1|X) = 1$, we assume $\theta_0 = [0, \ldots, 0]$.

We then use maximum likelihood estimation to obtain the log odds ratio estimates $\boldsymbol{\theta}_j = [\theta_{j1}, \theta_{j2}, \ldots, \theta_{jm}]$, $j = 1, \ldots, K$, for the $j$th case type in the polytomous logistic model in (1) from SAS proc logistic with the glogit link. Note that $\theta_{j1}$ in formula (1) above denotes the intercept term.

Expected prevalences for the cancer groups are then calculated as follow:

1. Fit model (1) to the SEER population with age, sex, year, and race/ethnicity as covariates X.
2. For each person in the DM cohort, plug their age, sex, year, and race and ethnicity into formula (1) with the parameters estimated from SEER. The probability of the reference group is obtained using $\mathrm{P}(Y_0 = 1|X) = 1 - P(Y = 1|X) - P(Y = 2|X) - \ldots . P(Y = K|X) = 1 - \sum_{i=1}^{K} \frac{exp(\theta_{i1}+\theta_{i2}X_1+\cdots\theta_{im}X_m)}{1+\sum_{k=1}^{K} exp(\theta_{k1}+\theta_{k2}X_1+\cdots\theta_{km}X_m x)}$

© 2025 Gadalla SM et al. JAMA Network Open.

So for each person we get K+1 probabilities.

3. Then, we summed these vectors of K+1 estimated probabilities for each person over all DM patients. That gives us the expected prevalences of the cancers in the K+1 categories.
4. We then take the ratio of these numbers with the observed numbers in each cancer category in the DM population, to get an observed over expected ratio.

© 2025 Gadalla SM et al. JAMA Network Open.
