# Supplementary material for: Spectrum of Cancers and Their Prognosis Among Patients With Myotonic Dystrophy
Source: JAMA Netw Open. 2025 Aug 13;8(8):e2526894. doi: 10.1001/jamanetworkopen.2025.26894 (PMC12351407; doi:10.1001/jamanetworkopen.2025.26894)
Supplement: Supplement 2. — Data Sharing Statement [file jamanetwopen-e2526894-s002.pdf]

Data Sharing Statement

Gadalla. Spectrum of Cancers and Their Prognosis Among Patients with Myotonic Dystrophy. *JAMA Netw Open*. Published August 13, 2025. doi:10.1001/jamanetworkopen.2025.26894

Data
Data available: No

Additional Information
Explanation for why data not available: The study used the SEER Medicare database which can be requested through the process described in here
https://healthcaredelivery.cancer.gov/seermedicare/obtain/requests.html
